# Enhanced Cytotoxic Effects of Cold Plasma Deposition of Topotecan: A Novel Approach for Local Cancer Drug Delivery to Glioblastoma Cells

**DOI:** 10.3390/cancers17020201

**Published:** 2025-01-09

**Authors:** Beatriz Pinheiro Lopes, Liam O’Neill, Paula Bourke, Daniela Boehm

**Affiliations:** 1 School of Chemical and Bioprocess Engineering, University College Dublin, D04 V1W8 Dublin, Ireland; 2 Sustainability and Health Research Hub and School of Food Science and Environmental Health, Technological University Dublin, D07 H6K8 Dublin, Ireland; 3 TheraDep Ltd., QUESTUM Innovation Centre, E91 V329 Clonmel, Ireland; 4 Plasma Research Group, School of Biosystems and Food Engineering, University College Dublin, D04 V1W8 Dublin, Ireland; 5 Conway Institute, University College Dublin, D04 V1W8 Dublin, Ireland

**Keywords:** glioblastoma, anticancer treatment, cold atmospheric plasma, plasma deposition, plasma-based technologies, topotecan, combination treatments

## Abstract

Background/Objectives: Despite the numerous advances in glioblastoma multiforme (GBM) treatment, GBM remains as the most malignant and aggressive form of brain cancer, characterized by a very poor outcome, highlighting the ongoing need for the development of new therapeutic strategies. A novel intervention using plasma-assisted local delivery of oncology drugs was developed to mediate the drug delivery, which might improve drug uptake and/or chemotherapeutic action. Topotecan (TPT), a water-soluble topoisomerase I inhibitor with major cytotoxic effects during the S-phase of the cell cycle, was selected as the candidate drug because despite its potent antitumor activity, the systemic administration to the brain is limited due to low crossing of the blood-brain barrier. For these reasons, TPT may be repurposed for local combined therapies. Methods: We aimed to explore options for the local treatment of GBM where systematic delivery is challenging, using a combination between plasma-based technologies and TPT on a human brain cancer cell line (U-251mg). Results: The evaluation of direct TPT plasma deposition using a helium plasma jet (J-Plasma, Apyx Medical) with a nebulizer onto U-251mg cells grown in 2D or 3D culture showed a reduction in the metabolic activity and cell mass and decreased long-term survival, indicating synergistic effects between the drug and the plasma treatment. The plasma-assisted approach was confirmed using temozolomide (TMZ) as a standard drug for glioblastoma treatment, as well as with two skin cancer cell lines. Conclusions: These results revealed a pathway for new combinations and approaches to local drug application for a range of cancers.

## Simple Summary

Glioblastoma multiforme (GBM) is a highly aggressive brain cancer with limited treatment options, highlighting the urgent need for novel therapeutic approaches, particularly those that can act locally. This study investigates a plasma-assisted method aimed at enhancing the local delivery of oncology drugs, focusing specifically on topotecan (TPT), a potent topoisomerase I inhibitor. Although TPT demonstrates potent antitumor activity, its use in systemic treatments is limited by its inability to effectively cross the blood–brain barrier, making it a promising candidate for local therapy. In this study, a nebulizer was attached to the J-Plasma^®^ device—a medically approved helium plasma jet—and used to directly deposit TPT onto GBM cells (U-251mg) grown in both 2D and 3D culture systems. This method led to a reduction in cell metabolic activity, mass, and survival, indicating enhanced drug uptake and therapeutic efficacy. Additionally, the standard GBM treatment temozolomide (TMZ) and two skin cancer cell lines were tested, further supporting the potential of plasma-based drug delivery. These findings open new avenues for innovative local therapies that could potentially extend to a variety of cancers.

## 1. Introduction

Glioblastoma multiforme (GBM) is a grade IV astrocytoma and one of the most malignant, aggressive, and common forms of primary brain tumour in adults. Despite years of efforts to combat this disease [1], few new therapies have proven effective against GBM due to its high resistance to treatments, primarily attributed to the blood–brain barrier (BBB) [1,2]. The current standard treatment for GBM remains as surgical resection or biopsy followed by adjuvant radiation and chemotherapy with temozolomide (TMZ) [3,4]. Achieving complete remission remains challenging, as approximately 80% of glioblastoma recurrences occur within or at the margin of the radiation field [5]. This emphasizes the importance of identifying and developing novel technologies that target the resection cavity to mitigate early tumour recurrence [1]. Localized approaches can take advantage of the unique characteristics of the BBB to limit the risk of systemic toxicity [6], thereby expanding the possibilities for chemotherapy combinations [7].

In this field of research, new locally administered therapies are being developed, including stereotactic injections, intra-arterial delivery, and convection-enhanced delivery [1,8,9]. These techniques also allow for the repurposing of established chemotherapeutics, which would not typically be used for GBM. This includes chemotherapeutics such as topotecan (TPT), a water-soluble topoisomerase I inhibitor that induces DNA damage, impacts the cell cycle, and disrupts cellular proliferation [8]. In preclinical models, TPT demonstrated potent antitumor activity with minimal impact on the normal brain, but its systemic use is limited due to high toxicity. This makes it a promising candidate for further investigation in local delivery methods [10].

Plasma deposition involves the nebulization of materials combined with a plasma treatment integrated in a single treatment to achieve the generation of a thin film [11]. Despite being a recent field, plasma deposition has been extensively researched for various biomedical applications, including biological collagen coatings [11,12], new therapeutic pharmaceutical coatings [13], antimicrobial coatings [14], and antibiotic/drug slow-releasing systems [15]. Studies have shown the benefits of plasma treatments in wound healing [16,17] and cancer therapy [18], leading to the development of a new plasma-based approach that aims to deliver oncology drugs directly to cancerous tissue. Especially for GBM, there is a lack of effective and life extending interventions, with current therapeutic strategies still depending on systemic drug delivery and surgery. Despite advancements in immuno-oncology, nanomedicine, and other BBB penetrating strategies [19], the clinical outcomes remain suboptimal due to challenges relating to tumour location and the blood–brain barrier, which hamper effective treatment options. For those reasons, the urgent challenge is to develop innovative delivery systems that are compatible with surgery, can overcome or bypass the BBB, and retain or enhance drug efficacy while minimizing adverse effects. The use of plasma-based therapies to target tumour margins directly, especially post-surgical resection, could potentially offer a less invasive and stressful treatment option with reduced side effects compared to systemic chemotherapy, while also reducing the financial burden associated with treatment [20] by mediating drug delivery and potentially enhancing drug uptake.

The main goal of this study is to explore plasma deposition as a new local chemotherapy delivery system for GBM and to validate the approach for other cancer types and oncology drugs. A clear set of objectives was addressed: to evaluate the effect of the direct plasma TPT deposition on a GBM cell line and on a more complex model system (3D spheroids) of GBM in comparison to wet-deposited TPT and plasma treatment; to determine the applicability of this approach to other cancer types by assessing the influence of direct TPT deposition onto a malignant melanoma and an epidermoid squamous carcinoma cell line model, and to determine the scope of depositing different oncology drugs by testing the plasma deposition of the GBM standard oncology drug TMZ.

## 2. Materials and Methods

### 2.1. J-Plasma^®^ APYX Medical Device for Topotecan and Temozolomide Deposition

**Figure 1:**
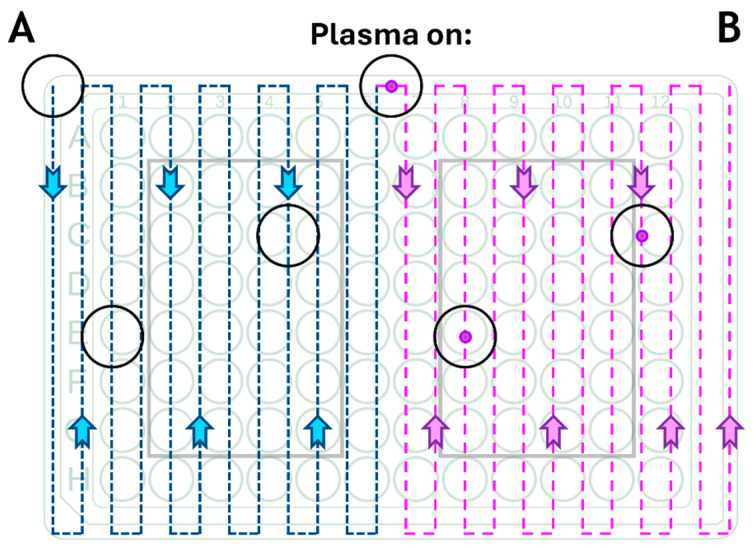
Schematic of the CNC raster pattern for deposition protocol. (**A**) TPT wet deposition protocol, where the plate was coated using only a constant flow of nebulized TPT and (**B**) TPT plasma deposition protocol, where the plasma discharge was combined with the nebulized TPT.

Plasma deposition was carried out using the Apyx Medical Ultimate^®^ Electro-surgical Generator, an FDA cleared (FDA: K192867) RF power supply designed to be used with a wide range of electro-surgical tools that has already entered the marketplace. When used with Apyx Medical’s J-Plasma hand piece (FDA: K183610), it can create a low temperature helium corona discharge. In this study, the J-Plasma hand piece was combined with a pneumatic nebuliser (T2100, Burgener Research, Mississauga, ON, Canada) connected to a syringe pump (NE-300 just infusion syringe pump, New Era Pump Systems, Farmingdale, NY, USA) to provide a constant flow of TPT/TMZ solution at 10 μL/min. The liquid was nebulized using a helium gas flow of approximately 2 slm. The plasma power was set on the Apyx Medical Ultimate generator at 40 W, and the helium flow was 4 slm. The combined unit consisted of an inert polymer block into which both the J-Plasma hand piece and the nebuliser could be inserted. This polymer block then directed the plasma discharge from the hand piece and the aerosol spray from the nebuliser into a confined acrylic tube (19 mm inner diameter × 35 mm length) in which the plasma and aerosol could interact before exiting the tube and being directed onto the target substrate. The coatings were deposited by moving the plasma unit over the desired target using a pre-programmed computer numerically controlled (CNC) system, using a raster pattern with a 4 mm step size and 1000 mm/second traverse speed (Figure 1).

**Figure 2:**
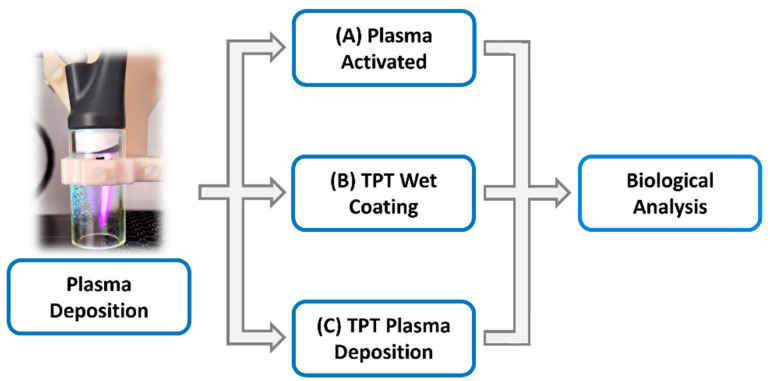
Schematic of the plasma deposition protocol whereby the plates were treated with different approaches. (A) Plasma-activated protocol, where the plate was treated only with plasma discharge; (B) TPT wet-coating protocol, where the plate was treated using only a constant flow of nebulized TPT; (C) TPT plasma deposition protocol, where the plasma discharge was combined with the nebulized TPT. Plates without any deposition treatment were used as control for all the biological experiments.

The traverse speed was adjusted in order to produce results with 1×, 3×, 6×, 9×, and 12× slower speed, in comparison with 1×, 3×, 6×, 9×, and 12× treatment cycles for 2D cell culture analysis. For treatment of cells grown in 3D, the traverse speed was set up to 10× slower. In order to produce equivalent coatings using a wet chemical spray approach, the same procedure was used, but the plasma was turned off (Figure 2). The deposition process presented an efficacy of around 50%, meaning that around 50% of the drug was lost either via evaporation or deposition onto the walls of the acrylic tube [21].

### 2.2. Cell Culture

#### 2.2.1. Two-Dimensional Cell Culture

The Human GBM cell line U-251mg (formerly known as U-373mg-CD14), the human malignant melanoma cell line A375, and the human epidermoid squamous carcinoma cell line A431 were obtained through the Sustainability and Health Research Hub, Technological University Dublin. Cells were cultured in Dulbecco’s modified eagle medium/nutrient mixture F-12 ham (DMEM/F12, Sigma-Aldrich, Arklow, Ireland) supplemented with 10% fetal bovine serum (FBS, Sigma-Aldrich, Arklow, Ireland) and 2mM L-glutamine (Sigma-Aldrich, Arklow, Ireland) in a humidified incubator at 37 °C with 5% CO_2_. Cells were routinely sub-cultured when 80% confluence was reached using 0.25% *w*/*v* trypsin solution. For each assay, cells at a density of 2.5 × 10^4^ cells/mL were plated in a 96-well (100 μL) plate and incubated overnight to allow for cell adhesion.

#### 2.2.2. Three-Dimensional Cell Culture

U-251mg human GBM cells were used to generate tumour spheroids. For spheroid analysis, 10,000 cells were seeded into each well of a 96-well round bottom plate, previously coated with the low attachment Biofloat^TM^ flex coating solution (faCellitate, Mannheim, Germany) in DMEM/F12 (Sigma-Aldrich, Arklow, Ireland) supplemented with 10% FBS (Sigma-Aldrich, Arklow, Ireland) and 2 mM L-glutamine (Sigma-Aldrich, Arklow, Ireland). The low attachment plates were centrifuged at 500× *g* for 5 min followed by incubation (37 °C, 5% CO_2_, 95% humidity). The spheroids were left to growth for 3 days before any experiments.

### 2.3. Cytotoxicity Evaluation

#### 2.3.1. Stock Solution Preparation

Both topotecan (TPT, Sigma-Aldrich, Arklow, Ireland) and temozolomide (TMZ, Fisher Scientific BVBA, Brussel, Belgium) were dissolved in dimethyl sulfoxide (DMSO) (Sigma-Aldrich, Arklow, Ireland) to achieve a final concentration of 47.5 mM and 100 mM and were stored at −20 °C. These stocks were subsequently used to make the working standard solutions both in DMSO and H_2_O as well as in media.

#### 2.3.2. Dose–Response Curves

Dose–response curves for plasma, wet- or plasma-deposited topotecan (TPT, Sigma-Aldrich, Arklow, Ireland), and temozolomide (TMZ, Fisher Scientific BVBA, Brussel, Belgium) were established for U-251mg 2D cultures, while a dose–response curve for TPT was performed for both the A431 and A375 cell lines in 2D culture and U-251mg cell line in 3D culture.

Existing media was replaced with fresh media right before cells were treated with either plasma, wet- or plasma-deposited TPT, or TMZ, and cells were incubated for 72 h post-treatment. Dose–response curves were established for the plasma treatment by reducing the traverse speed (1×, 3×, 6×, 9×, and 12× slower) or increasing the number of treatments (traverses) applied (1×, 3×, 6×, 9×, and 12×). For the TPT dose–response curves, final concentrations of 137.08, 68.54, 51.40, 34.27, 29.13, 23.99, 18,85, 13,71, 8.57, 4.28, 2.14, and 1.07 nM were used for the wet or plasma deposition onto 2D culture. A range of concentrations of TPT of 237.50, 118.75, 59.38, 29.69, 14.84, 7.42, 3.71, 1.86, 0.93, 0.46, 0.23, 0.12, 0.06, 0.03, 0.01, 0.007, 0.004, 0.002, and 0.001 μM were used for U-251mg in 3D culture. For the TMZ dose–response curve, final concentrations of 1000.00, 500.00, 250.00, 125.00, 62.50, 31.25, 15.62, 7.81, 3.91, and 1.95 μM were used for U-251mg in 2D culture. TPT concentrations of 14,843.75, 7421.88, 3710.94, 1855.47, 927.73, 463.87, 231.93, 115.97, 57.98, 28.99, 14.50, 7.25, 3.62, 1.81, 0.91, 0.45, 0.23, 0.11, and 0.06 μM were used for A375 and A431 in 2D culture.

#### 2.3.3. Combined Treatment

Based on individual treatment dose–response curves, different conditions around the individual IC50 value were selected for further combinatorial studies. The overall parameters were selected based on previous work [22], with an incubation time of 72 h and a final volume in the well of 100 μL for all the conditions.

For the plasma treatments, the speed selected was 1× and 3× slower for 2D culture treatments with TPT and TMZ, respectively, and 10× slower for 3D culture treatments. For the TPT treatment, final well concentrations of 12.00 nM, 13.71 nM, and 24.00 nM were selected for U-251mg cell line 2D culture, while final well concentrations of 59.3 μM or 5.93 μM were selected for U-251mg cell line 3D culture. A total of 30 μM was chosen as the final well concentration for U-251mg cell line TMZ treatment. A total of 27.90 nM and 55.90 nM were chosen as the TPT final well concentrations for both the A375 and A431 cell lines.

### 2.4. Cell Viability Assays

#### 2.4.1. Resazurin/Alamar Blue Assay

Cell metabolic activity was analyzed using the resazurin assay (Sigma-Aldrich, Arklow, Ireland) as described before [22]. This assay is based on the reduction in the oxidized blue dye (resazurin) to a pink dye (resorufin) by metabolically active cells (live cells). After 72 h exposure time, cells were washed once with sterile phosphate-buffered saline (PBS, Sigma-Aldrich, Arklow, Ireland) and incubated for 2 h or 24 h at 37 °C with 100 μL of resazurin (final concentration 8 μg/mL) in the cell culture medium, without or with FBS, for 2D culture and 3D culture, respectively. Absorbance was monitored via a SpectraMax iD3-3211 multi-mode microplate reader (Molecular Devices, Berkshire, UK) at 570 nm and 600 nm. The results are expressed as a percentage of the metabolic activity normalized to control cells.

#### 2.4.2. Crystal Violet Staining

Cell mass was assessed via crystal violet colorimetric growth assay as described elsewhere [22]. After 72 h exposure time, cells were washed once with PBS, and adherent cells were fixed with 70% methanol (Sigma-Aldrich, Arklow, Ireland) for 1 min, and then stained with 0.2% crystal violet solution (Sigma-Aldrich, Arklow, Ireland) for 10 min. Excess stain was rinsed off with water, and plates were left to air-dry overnight. The dye bound to the adherent cells was re-solubilised with 10% acetic acid (Sigma-Aldrich, Arklow, Ireland) and absorbance was measured at 600 nm using a SpectraMax iD3-3211 multi-mode microplate reader (Molecular Devices, Berkshire, UK). Cell mass is expressed as a percentage normalized to control cells.

#### 2.4.3. CellTiter-Glo^®^ 3D

Cell viability in 3D spheroids was evaluated via luminescence, employing ATP as an indicator of metabolically active cells. The assay reagent has a robust lytic capacity, penetrating the spheroids and exposing the intracellular ATP to a thermostable luciferase. The luciferase reaction results in the generation of a luminescent signal proportional to the amount of ATP present, which is directly proportional to the number of viable cells. After 3, 6, or 8 days of incubation, the spheroids were transferred in 50 μL of media to a 96-well white flat bottom plate (ThermoFisher, Dublin, Ireland). A total of 50 μL of CellTiter-Glo^®^ 3D reagent (Promega, Chilworth, Hampshire, Great Britain) was then added to each well, and the plate was mixed for 5 min on a shaker. After that, the cells were incubated 25 min at room temperature, and luminescence was monitored using a SpectraMax iD3-3211 multi-mode microplate reader (Molecular Devices, Berkshire, UK). The results are expressed as a percentage of metabolic activity normalized to control cells.

### 2.5. Combination Index

To determine the nature of interaction of the combination of TPT and plasma in the U-251mg cell line, the combination index (CI) value was calculated as follows:

(1) $$CI=\left(C_{TPT}/I_{TPT}\right)+\left(C_{Plasma}/I_{Plasma}\right)$$

In this case, *C_TPT_* and *C_Plasma_* refer to the concentrations of TPT and plasma when in combination treatment, while *I_TPT_* and *I_Plasma_* refer to the concentrations of TPT and plasma in individual treatments that achieved the same effect as the combination. In general, CI < 0.8 indicates synergism, CI = 0.8–1.2 indicates additive, and CI > 1.2 refers to antagonism [23].

### 2.6. Evaluation of Cell Survival Clonogenic Assay

A cell survival assay was performed, which evaluated the ability of a single cell to form a colony. After 72 h incubation post-plasma, wet or plasma TPT deposition, cells were harvested, and 500 cells per condition were plated in 6-well plates in a total volume of 3 mL of complete medium. The medium was renewed after 7 days and after 14 days; colony formation was assessed by staining with the crystal violet solution (as described above in Section 2.4.2). The survival factor was calculated as follows [24]:

(2) $$Survival\;factor\;\left(\%\right)=\frac{absorbance\;of\;treated\;cells}{absorbance\;of\;control\;cells}\times 100$$

### 2.7. Statistical Analysis

All the experiments were performed at least three independent times. Prism version 8.0.1, (GraphPad Software, San Diego, CA, USA) was used to carry out curve fitting and statistical analysis. Dose–response curves were measured using nonlinear regression. Data are presented as the mean and standard deviation (SD). Multiple comparison analyses were performed using two-way ANOVA with Tukey’s post-test. The significance is indicated in figures as * *p* < 0.05, ** *p* < 0.01, *** *p* < 0.001, and **** *p* < 0.0001.

## 3. Results

### 3.1. Direct Plasma Treatment and TPT Combinations Decrease the Survival Rate of U-251mg Glioblastoma Cell Line

**Figure 3:**
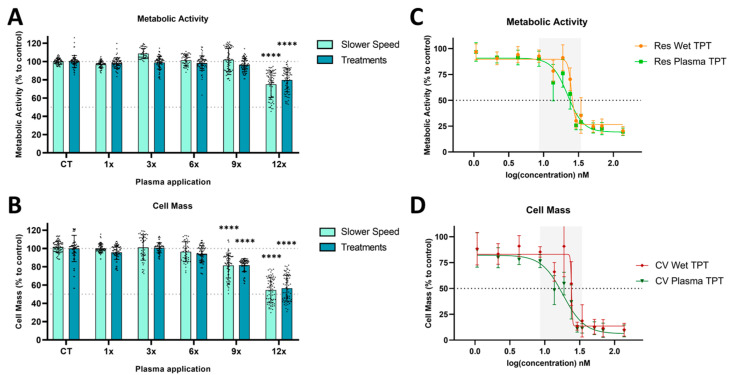
Dose–response effect of direct plasma treatment, wet-, or plasma-deposited TPT treatments in U-251mg glioblastoma cell line. Dose–response curves were obtained based on metabolic activity (resazurin assay) and cell mass (crystal violet assay). Dose–response of (**A**,**B**) direct plasma treatment and (**C**,**D**) wet- or plasma-deposited TPT treatments. Results are presented as mean ± SD and as comparisons to control. Statistical significance is shown in comparison to control and represented as **** *p* < 0.0001.

Cells were treated with either direct plasma, wet TPT (wet-deposited TPT using nebulizer), or plasma TPT (plasma-deposited TPT). Subsequently, the metabolic activity (resazurin) and cell mass (crystal violet) were evaluated (Figure 3). The results demonstrated that direct plasma treatment produced comparable cytotoxic effects when considering multiple treatments or slower speed (Figure 3A,B). However, even after 12 treatment cycles/12× slower speed, the plasma treatment was only able to reduce the cell viability to around 50% (preventing the proper calculation of the true IC50 value). A comparison between the effects of wet TPT and plasma TPT highlighted a potential area of interest for deposition application (Figure 3C,D—grey area). Additionally, the calculated IC50 values revealed that wet TPT requires approximately 25% higher concentration of TPT deposited to achieve a similar effect to that of plasma TPT (Table 1).

Since it was not possible to calculate an IC50 for plasma treatment, the IC36 (corresponding to around 64% of cell viability) was used to calculate the combination index (CI) of the combined treatments. The CI value obtained was 0.59, corresponding to synergism [23], indicating that plasma TPT could be a good approach for further investigation.

### 3.2. TPT Combinations Have an Antiproliferative Effect in U-251mg Glioblastoma Cell Line

**Figure 4:**
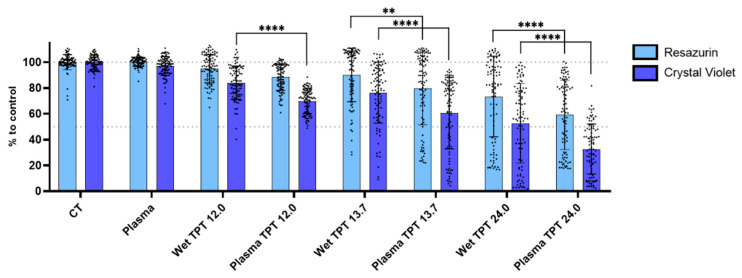
Wet- or plasma-deposited TPT treatments reduce the metabolic activity and cell mass in the U-251mg glioblastoma cell line. Quantification of the combinatorial effect was performed by resazurin and crystal violet assays 72 h after treatment with plasma, wet- or plasma-deposited TPT. Results are presented as mean ± SD in comparison to control. Statistical significance is shown in comparison to wet-deposited TPT treatment and represented as ** *p* < 0.01; **** *p* < 0.0001.

For the combinatory effects of the plasma TPT, we selected 1× treatment cycle for plasma treatment and 12.00, 13.71, and 24.00 nM for the TPT concentrations (TPT 12.0, TPT 13.7, and TPT 24.0). The metabolic activity and cell mass were evaluated 72 h after treatment with plasma, wet TPT, and plasma TPT (Figure 4). Both metabolic activity and cell mass decreased with wet TPT 12.0 (94.97 ± 10.84% and 84.23 ± 12.82%), wet TPT 13.7 (90.17 ± 20.67% and 76.42 ± 23.65%), and wet TPT 24.0 (73.45 ± 31.25% and 52.69 ± 30.69%). The plasma deposition treatments promoted a significantly enhanced effect (*p* < 0.01) when compared to the wet deposition (88.52 ± 10.16% and 69.78 ± 9.59%) for TPT 12.0, (79.93 ± 28.38% and 60.52 ± 27.56%) for TPT 13.71, and (59.23 ± 26.87% and 32.69 ± 19.26%) for TPT 24.0.

### 3.3. TPT Treatments Decrease the Survival Rate of Melanoma and Squamous Carcinoma Cell Lines

To evaluate the potential applicability of the plasma deposition technique across diverse cancer types, two skin cancer models—malignant melanoma (A375 cell line) and epidermoid squamous carcinoma (A431 cell line)—were utilized in this study. Considering that biological factors such as cell type, cancer type, and cell culture medium can influence the plasma treatment [25], all cancer cell lines were previously sub-cultured in DMEM to eliminate one of these variables.

**Figure 5:**
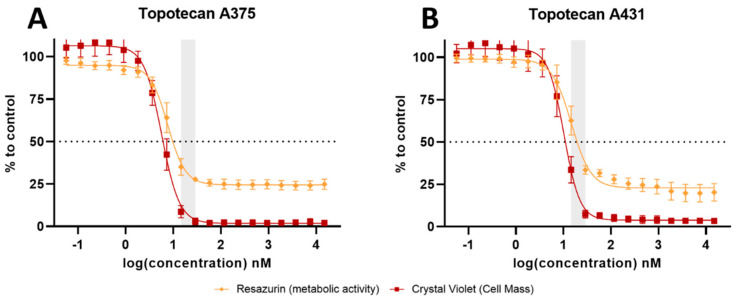
Dose–response effect of TPT individual treatment in melanoma and squamous carcinoma cell lines. Dose–response curves for metabolic activity and cell mass were obtained by resazurin and crystal violet assays. IC50 curves of TPT treatment alone (**A**) in the A375 melanoma cell line and (**B**) in the A431 squamous carcinoma cell line. Results are presented as mean ± SD and as comparison to control.

The A375 and A431 cells were exposed to increasing concentrations of TPT, and then, the metabolic activity (resazurin) and cell mass (crystal violet) were assessed to identify the optimal concentration range for potential application in plasma deposition (Figure 5). The estimated IC50 values indicated that the A375 cells exhibited lower sensitivity to TPT treatment compared to the A431 cells, with an approximately double IC50 value observed for the A375 cells (Table 2).

**Figure 6:**
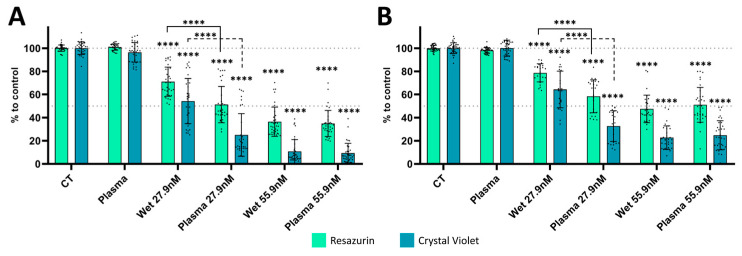
Wet- or plasma-deposited TPT treatments reduce the metabolic activity and cell mass in the A375 melanoma and A431 squamous carcinoma cell lines. Quantification of the combinatorial effect was performed using resazurin and crystal violet assays 72 h after treatment with plasma, wet-, or plasma-deposited TPT in (**A**) the A375 melanoma cell line and in (**B**) the A431 squamous carcinoma cell line. Results are presented as mean ± SD in comparison to control. Statistical significance (filled line for resazurin and dotted line for crystal violet) is shown in comparison to CT (symbol *) or wet-deposited TPT treatment (symbol #) and represented as **** *p* < 0.0001.

After some optimization process, TPT concentrations of 27.9 nM and 55.9 nM (TPT 27.9 and TPT 55.9) were chosen for the deposition protocol (Figure 6). The metabolic activity and cell mass were evaluated 72 h after treatment with plasma, wet TPT, and plasma TPT (Figure 6). The plasma treatment selected for this experiment was 1× treatment cycle, the same used for the treatment of the U-251mg cell line, without impacting the metabolic activity or cell mass of the A375 and A431 cells (Figure 6). This treatment cycle was selected to avoid multiple cycles, since there is some evidence that the A375 cell line can indeed acquire resistance to plasma treatment cycles [25].

For the A375 cell line both metabolic activity and cell mass decreased with wet TPT 27.9 (71.05 ± 12.59% and 59.78 ± 18.38%) and wet TPT 55.9 (36.41 ± 12.70% and 12.03 ± 11.53%). The plasma deposition treatment promoted a significantly enhanced effect (*p* < 0.0001) when compared to the wet deposition with TPT 27.9, reducing both metabolic activity (51.23 ± 15.68%) and cell mass (28.19 ± 20.67%) (Figure 6A). For higher concentrations of TPT, no statistical significance was observed between the two treatments with TPT 55.9.

A similar behaviour was observed for the A431 cell line where the metabolic activity and cell mass were decreased, respectively, via wet deposition with TPT 27.9 (78.62 ± 7.82% and 72.47 ± 12.637%) and TPT 55.9 (47.56 ± 11.88% and 24.09 ± 11.10%) (Figure 6B). Plasma deposition treatment achieved significantly higher reduction (*p* < 0.0001) than the wet deposition with TPT 27.9 (58.42 ± 14.21%) for metabolic activity and (24.62 ± 9.97%) for cell mass. For treatments with TPT 55.9, no statistical difference was observed between wet and plasma deposition treatments.

### 3.4. Plasma Deposition Does Not Limit the Efficacy of TMZ in U-251mg Glioblastoma Cell Line, Displaying a More Consistent Cytotoxic Effect than the Wet TMZ Coating

In order to explore the potential of the plasma deposition process, we not only conducted tests on different cell lines (glioblastoma and melanoma/squamous carcinoma 2D models) but also examined the effectiveness of the process using a different oncology drug (TMZ which is often used as a first-line treatment for GBM).

**Figure 7:**
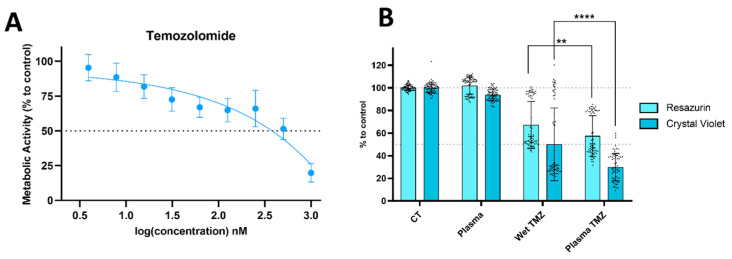
TMZ plasma deposition presented a more consistent cytotoxic effect in the U-251mg cell line than the wet coating. These results were obtained 72 h after treatment with TMZ, direct plasma, wet-, or plasma-deposited TMZ, via resazurin and crystal violet assays. (**A**) Dose–response curve for TMZ treatment alone and (**B**) quantification of the combinatorial effect in glioblastoma cells using resazurin and crystal violet assays 72 h after treatment with plasma (3× slower speed), wet-, or plasma-deposited TMZ (30 µM). Results are presented as mean ± SD and as comparison to control. Statistical significance is represented as ** *p* < 0.01; **** *p* < 0.0001.

The U-251mg cells were treated with increasing concentrations of temozolomide (TMZ), and then, the metabolic activity (resazurin) was evaluated to determine the IC25 value for a potential application in plasma deposition (Figure 7A). A TMZ concentration of 30 μM was chosen for the deposition protocol based on the dose–response curve (Figure 7B). Given the solubility limit of TMZ, a 3× slower plasma treatment speed was selected for this experiment, without impacting the metabolic activity or cell mass of the U-251mg cells (Figure 7B). Both metabolic activity and cell mass decreased with wet TMZ (67.24 ± 20.81% and 50.09 ± 32.20%), with the plasma deposition treatments showing again an enhanced effect (57.53 ± 17.94% and 29.89 ± 12.16%) when in comparison (*p* < 0.01) to the wet deposition.

### 3.5. TPT Treatment Alone Presents Cytotoxicity Toward U-251mg Glioblastoma Cell Line Spheroids in a Dose-Dependent Manner

**Figure 8:**
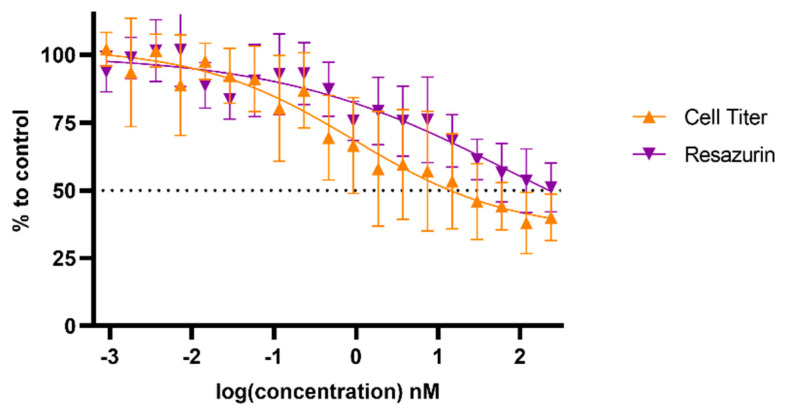
Metabolic activity dose–response effect of TPT treatment alone in the U-251mg glioblastoma cell line spheroids. Dose–response curves were obtained via resazurin and CellTiter-Glo^®^ 3D assays 72 h post-treatment. Results are presented as mean ± SD and as comparisons to control.

The U-251mg cell line spheroids were cultured for 3 days prior to treatment, with media replacement just before the addition of increasing concentrations of TPT. The metabolic activity was evaluated 72 h post-treatment, either via resazurin or CellTiter-Glo^®^ 3D assays (Figure 8). Based on the dose–response curve for the TPT, it was possible to determine the range of concentrations to be used for the TPT plasma deposition onto spheroids.

### 3.6. TPT Direct Plasma Deposition onto U-251mg Spheroids Induces Cell Death, Even with Lower Concentrations

**Figure 9:**
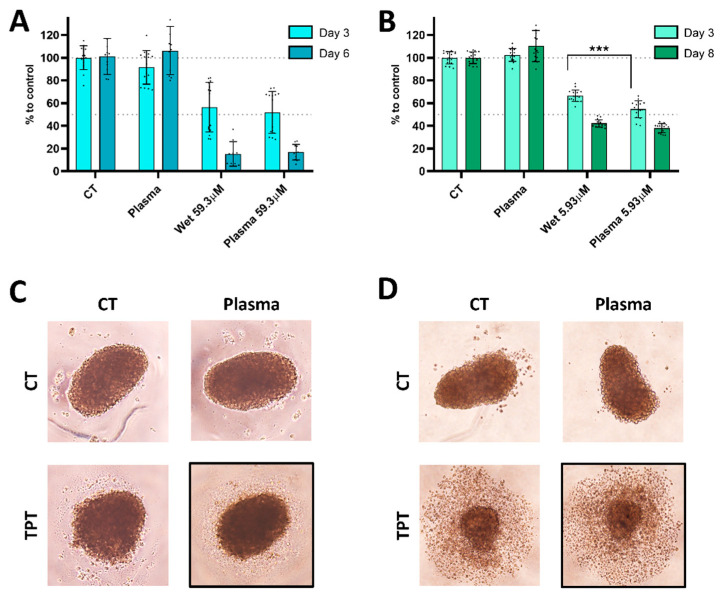
TPT plasma deposition treatment promotes cytotoxic effect in U-251mg spheroids. Spheroids were treated either with direct plasma, wet-, or plasma-deposited TPT and incubated for 72 h. After that, the media was replaced with non-treated media, and the spheroids were cultured for another 72 h or 144 h. Results were obtained via CellTiter-Glo^®^ 3D assay. The metabolic activity was quantified in relation to control for TPT concentrations of (**A**) 59.3 μM and (**B**) 5.93 μM on each well. Representative images of the spheroids treated with 59.3 μM on the well after 3 days (**C**) or 6 days (**D**) with medium changed at day 3. Results are presented as mean ± SD and as comparisons to control. Statistical significance is represented as *** *p* < 0.001.

In order to assess the effectiveness of plasma deposition on spheroids, TPT concentrations of 59.3 μM (approximately IC50) and 5.93 μM (approximately IC40) were chosen for evaluation (Figure 9). These concentrations were selected based on a serial dilution (1:10). Due to the solubility limit of TPT, a plasma treatment at a speed 10× slower was chosen, without impacting the metabolic activity of the U-251mg spheroids. The metabolic activity was measured at 3 and 6 days after treatment with 59.3 μM (Figure 9A) and at 3 and 8 days after treatment with 5.93 μM (Figure 9B). By using the two different TPT concentrations (59.3 and 5.93 μM), it was possible to enquire about a possible prolonged effect of the plasma deposition. After 3 days of treatment, the media was removed, and the spheroids were allowed to recover in a TPT-free medium.

The treatments with 59.3 μM of TPT resulted in a decrease in metabolic activity at day 3, with wet TPT 59.3 showing a reduction to 56.57% ± 21.93 and plasma TPT 59.3 to 51.80% ± 18.56. By day 6, the metabolic activity further decreased to 15.39% ± 10.18 with wet TPT 59.3 and to 16.78% ± 6.51 with plasma TPT 59.3 (Figure 9A), with no significant statistical difference between the two treatments. The images of spheroids treated with wet or plasma TPT 59.3 did not show any noticeable distinction between wet and plasma deposition, after 3 or 6 days, respectively (Figure 9C,D). When the TPT concentration was reduced 10-fold, the metabolic activity exhibited a decrease 3 days after treatment with wet and plasma TPT 5.93 at 66.53% ± 4.45 and 54.68% ± 7.83, respectively, showing a significantly stronger reduction in plasma TPT treated samples. By day 8, the metabolic activity remained around 42.19% ± 3.52 and 37.97% ± 4.11 (Figure 9B).

Cells treated with the higher TPT concentration started to die (after day 3) and could not recover, leading to continuous death until day 6. In contrast, cells treated with lower TPT concentration, experienced cell death after day 3, but gradually recovered until day 8, resulting in a lower cell death ratio (Figure 9A,B).

### 3.7. TPT Plasma Deposition Treatments Have a Long-Term Antiproliferative Effect in U-251mg Spheroids

In addition to assessing the short-term antiproliferative effects, we also investigated the impact on long-term survival post-treatment. To achieve this, three days following treatment, the spheroids were collected and dissociated to obtain a single-cell suspension (containing cells which survived plasma/TPT treatments). These cells were then allowed to recover in an untreated/TPT-free medium.

**Figure 10:**
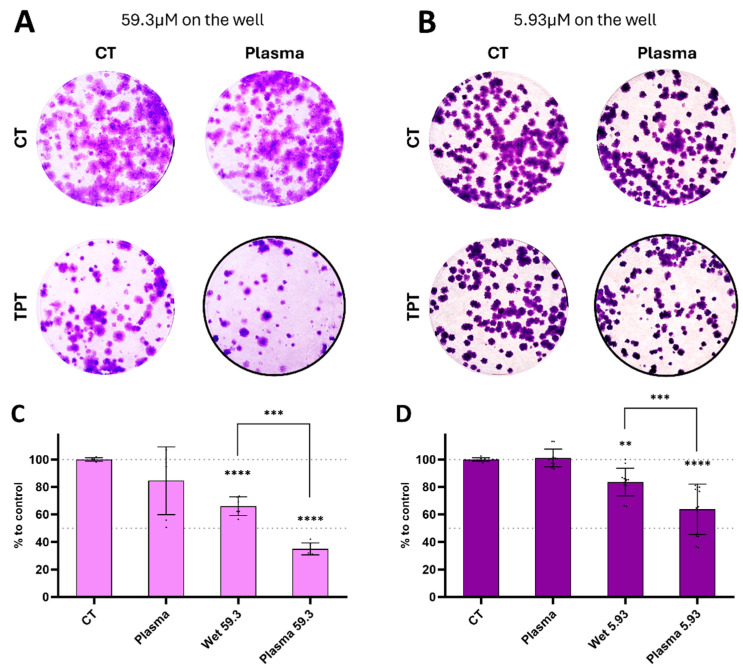
TPT plasma deposition treatment decreases long-term survival of the U-251mg spheroids. The spheroids were treated either with direct plasma, wet-, or plasma-deposited TPT, incubated for 72 h and mechanically dissociated into a single-cell suspension in fresh media. Colony formation was evaluated after 14 days. Representative images of the colonies formed 14 days after the end of treatments with (**A**) 59.3 μM and (**B**) 5.93 μM on the well. Quantification of cell mass in relation to control with (**C**) 59.3 μM and (**D**) 5.93 μM on the well. Results are presented as mean ± SD and as comparisons to control. Statistical significance is represented as ** *p* < 0.01, *** *p* < 0.001, and **** *p* < 0.0001.

The clonogenic assay results indicated a reduction in both colony formation and colony size, particularly evident with the combination treatment, even at lower concentrations of TPT (Figure 10A,B). The total cell mass showed a decrease following treatment with wet TPT 59.3 (66.03% ± 6.74), plasma TPT 59.3 (35.01% ± 4.37), wet TPT 5.93 (83.66% ± 10.03), and plasma TPT 5.93 (63.75% ± 18.34), as compared to the controls (Figure 10C,D).

## 4. Discussion

Current therapeutic strategies often depend on systemic drug delivery, resulting in considerable side effects and systemic toxicity, while also failing to achieve the desired tumour control. Therefore, the urgent challenge is to develop innovative delivery systems that are compatible with surgery and to retain or enhance drug efficacy but also minimize adverse effects by targeting tumour margins directly, especially post-surgical resection. Furthermore, cancer therapy faces increasing development resistance to traditional treatments. While new drug advancements seem promising, they often require years to obtain regulatory approval and come with significant financial and time investments as well as introduce additional risks [26]. Hence, drug repurposing (the identification of new applications for existing and approved drugs) is gathering increased attention from the scientific community. This approach can help reduce costs and save valuable time given that most of the drug’s associated risks are already well documented [26]. TPT is known for its high toxicities, which are poorly tolerated when administered systemically, leading to limited antitumor effects [27]. However, in preclinical models, TPT has shown potent antitumor activity with minimal impact on normal brain tissue when used as a local therapy. This promising profile places TPT as a strong candidate for further exploration in this field [10,28], especially considering its recent identification as a potential candidate for drug repurposing in GBM therapy, with tests performed in various cell lines [29]. TPT targets proliferating cells in the S-phase of the cell cycle, and since only a small percentage of glial cells undergo division and at a slower rate than cancer cells [30], it may be assumed that normal cells would be less effected than cancer cells. However, further comparative investigation applying normal–cancer cell pairs is required to support such a statement. Also, being a local approach, the localized toxic effects in situ should be lower when compared to standard treatments (especially systemic chemotherapy).

The repurposing of oncology drugs, such as topotecan, for local administration is therefore also being investigated to reduce the side effects associated and eliminate systemic toxicity [8,9,10,30]. Cold plasma therapy is emerging as an innovative treatment with a range of delivery modalities, with the potential to exhibit synergistic effects when combined with chemotherapeutic agents [31,32,33]. For that reason, a plasma deposition approach using the J-Plasma device (FDA-approved medical plasma device) was tested and validated as a new local chemotherapy delivery system (repurposing of TPT and TMZ) for GBM and skin cancers models.

The results presented here showcase the potential of utilizing the chemotherapeutic drug TPT in a plasma deposition process. The outcomes suggest that plasma deposition does not compromise the efficacy of TPT in glioblastoma cells and reveal a synergistic effect, characterized by a more consistent cytotoxic impact compared to wet TPT coating (Figure 4). The variability observed in the metabolic activity and cell mass results from the deposition treatments may be associated with TPT’s mechanism of action. TPT is a topoisomerase I (Top I) inhibitor, disrupting the S-phase of the cell cycle and cellular metabolic profile [8]. Consequently, the outcomes may vary based on the number of cells in the S-phase of the cell cycle at the time of TPT deposition.

The decision to validate the plasma deposition approach using a skin cancer model derives from two primary considerations. Firstly, the skin’s external nature makes it immediately accessible for treatment with the J-Plasma system under examination, particularly from a clinical perspective. Secondly, the extensive body of research on plasma therapies for skin cancer supported this choice. Skin cancer is one of the most prevalent tumours worldwide [34], with malignant melanoma and squamous cell carcinoma representing the most lethal forms [35]. Cold atmospheric plasma applications are under investigation as adjunctive therapies or delivery mechanisms for skin cancers [35]. Studies have shown that plasma-activated medium in combination with small molecules can exhibit a synergistic effect on human skin cancer cells [34]. Direct plasma treatment of skin cancer cells has been found to enhance natural killer cell activity, thereby promoting tumour cell death [35]. As mentioned previously, the variability in responses to TPT observed also for the A375 and A431 cell lines may be associated with the number of cells in the S-phase of the cell cycle at the time of TPT deposition, which will impact the outcome of the deposition protocol. Our results indicate that the plasma deposition protocol remains effective when utilized with skin cancer models (Figure 6). This method has the potential to facilitate the implementation of oncology drug deposition protocols across a broader spectrum of cancers, expanding its applicability. Nonetheless, further evaluation of the deposition effects on a wider variety of cell lines is still needed.

Focusing on the glioblastoma model, we selected temozolomide (TMZ) as an additional drug for evaluation, given its common use as a first-line treatment for glioblastoma. The established mechanism of action of TMZ involves the methylation of guanines during DNA replication. This leads to the formation of mismatched pairs (methyl-guanine paired with thymine instead of cytosine), inducing DNA damage and subsequent cell cycle arrest at the G2/M transition phase, followed by apoptotic cell death [36]. Furthermore, there is ongoing research investigating possible combinations between TMZ and plasma treatments, with promising results [37,38,39,40]. Similar to the findings observed with TPT deposition, plasma deposition of TMZ demonstrated a more consistent effect in comparison to wet deposition (Figure 7). These results suggest that the plasma deposition protocol can be employed with various oncology drugs, opening doors for the exploration of additional combinations with new molecules for either chemotherapy or immunotherapy purposes. In fact, some experimental small molecules [41] and copper-dependent bactericidal antibiotics [42] are being studied for combinations with plasma, and they could become a good fit for further investigation with plasma deposition.

Established glioblastoma cell lines provide convenient models for the rapid screening of drug combinations and preliminary results [43]. However, while these models offer versatility, low laboratory costs, high standardization, and reproducibility, the 2D glioblastoma cell line model lacks the complexity and suitability required for high-throughput analyses [36]. To address this limitation, we utilized a U-251mg 3D cell culture model, as previously described [2], to facilitate cell-to-cell interactions in all three dimensions, thereby introducing a more complex spatial organization. This model also provides a middle ground between cell lines and glioblastoma stem-like cells [44], allowing us to replicate the diffusion limits of nutrients, oxygen, and signalling molecules commonly observed in the micro-environment of in vivo tumours.

Plasma deposition treatments on spheroids showed a reduction in metabolic activity in short-term evaluation with an increase in the cell death after that. Those results were validated by the detached cells observed surrounding the spheroid. Collectively, the cells that survived the combined treatments exhibited a loss of proliferation capability and failed to generate new colonies to the same extent as the controls, particularly evident in the plasma deposition scenario (Figure 10). These findings, consistent with previously obtained results [22], suggest that the surviving cells experienced an arrest in cell cycle progression, with this arrest appearing to persist in time. These findings illustrate that plasma deposition treatments not only exhibit cytotoxic effects on cancer cells but also impact the reproductive capacity of surviving cells by inhibiting their ability to undergo unlimited division and form colonies. Cancer stem cells (CSCs) form a unique subpopulation within tumours, distinguished by their self-renewing capability, which reinforces tumour development, persistence, and recurrence, especially in cases of glioblastoma [43]. CSCs also enhance tumour cell heterogeneity and are key players in conferring resistance to both chemotherapy and radiotherapy [45]. These results are of particular importance, especially considering that glioblastoma recurrence commonly occurs in proximity to the resection site. Localized plasma deposition presents a unique opportunity to deliver high doses of chemotherapeutic agents to this area, targeting residual cells post-resection procedure.

The findings presented here underscore the significant potential of the plasma deposition protocol, not only for TPT application in GBM but also for exploring combinations with various oncology drugs to address a wider range of cancer types. While cancer cell line models offer versatility and convenience, they often fall short in terms of complexity, spatial organization, and cell-to-cell interactions. Future studies should incorporate more complex 3D culture systems, by co-culturing multiple cell lines (normal and cancerous) and integrating the tumour micro-environment and microbiome. Utilizing in ovo or in vivo models and employing a range of biomarkers could provide valuable insights into the effects of these factors on the development of clinical therapies prior to the Phase 1 trial process.

While the initial findings are promising, extensive further research as outlined above is required to fully understand the implications of J-plasma treatment on topotecan and its potential as a targeted cancer therapy.

## 5. Conclusions

In summary, our findings demonstrate that plasma deposition of TPT effectively reduces the metabolic activity and cell mass of the U-251mg glioblastoma cell line as well as the A375 malignant melanoma and A431 epidermoid squamous carcinoma cell lines in a synergistic way, exhibiting a more consistent effect compared to wet-coated TPT. Similarly, the plasma deposition of TMZ onto the U-251mg glioblastoma cell line produced comparable results, suggesting potential for other combinatorial approaches in plasma deposition applications. The results from more complex models (spheroids) also indicate that the plasma deposition of TPT induces a cytotoxic effect in short-term growth (after 72 h) and an antiproliferative effect on long-term survival (after 14 days).

The results presented here support the potential use of plasma deposition as an improved delivery system for oncology drugs, specifically for localized application on tumour margins. This approach could enhance local efficacy and potentially reduce the associated side effects. The hope is that the deposition protocol could also be exploited for other treatments, with different classes of drugs being deposited, such as antibiotics, dressings, and even immunotherapy drugs.

## Figures and Tables

**Table 1:** IC50 values for wet- or plasma-deposited TPT treatments in U-251mg glioblastoma cell line.

IC50 Value	Resazurin (R^2^)	Crystal Violet (R^2^)
Wet TPT	25.16 nM (0.8781)	24.14 nM (0.8143)
Plasma TPT	22.08 nM (0.8468)	18.16 nM (0.8471)

**Table 2:** IC50 values for TPT treatment in the A375 melanoma and A431 squamous carcinoma cell lines.

IC50 Value	Resazurin (R^2^)	Crystal Violet (R^2^)
A375	7.65 nM (0.9876)	5.68 nM (0.9891)
A431	14.70 nM (0.9803)	10.27 nM (0.9848)

## Data Availability

All data generated or analyzed during this study are included in this published article and are available from the corresponding author upon reasonable request.
